# Cancer Curers

**Published:** 1890-09-20

**Authors:** 


### The Doctor's Pulpit.

### CANCER CURERS.

Dr. Kennedy, a believer in Count Mattei, has an article in this month's *National Review* in which he sets forth the facts of a case of cancer cure; and challenges the medical profession in general, and Dr. Herbert Snow in particular, to produce as good results in similar cases. This challenge will be held by large numbers of people to be proof positive of the uselessness of the "knife cure" for cancer, and equally demonstrated certainty of the value of Count Mattei's methods of treatment. Now, it is easy to laugh at Dr. Kennedy, and Lady Paget, and Count Mattei; and it is also easy to treat the whole race of cancer curers and unqualified practitioners with contempt. But the man who has a kindly disposition towards all his fellow men and a wholesome desire to prevent them breaking their necks when he sees them tearing along at full speed towards a precipice, uses the weapon of contempt very sparingly. He knows well how little rational mankind is in the mass, and how very stupid and prejudiced even clever people are on a good many of the subjects which come up for consideration in life. We, therefore, being anxious that our non-professional readers should have the means of forming reasonable judgments on the new method of curing cancer, venture to offer one or two facts and arguments for their consideration.

Nine or ten years ago a medical man who held the position of hospital surgeon and lecturer to medical students at a well-known hospital, startled the medical profession by publishing accounts of a number of cases of internal cancer which he said had been cured by a particular drug. The reception given to those accounts by medical men generally showed well the attitude of mind of the profession towards new ideas and new methods of treatment. Practically every physician and surgeon in the country, from the highest to the lowest, declared his readiness to try the new treatment, and his fervent hope that it might prove as successful in other hands as it was said to have done in those of the original discoverer. The present writer tried the drug in several well-marked cases of cancer which were at various stages of development. The story of one patient was particularly interesting and sad. It was that of a lady, a little over fifty years of age, who had an exceedingly happy home and a devoted husband and children. Before the commencement of her disease she had had excellent health, and had looked almost ten years younger than her age. The advent of the cancer was a terrible blow to the whole family. They would have sacrificed all they possessed to obtain a cure. Sir James Paget, Sir Joseph Lister, and other eminent practitioners were seen in turn; but in spite of all efforts the disease progressed.

It was at this juncture that the new treatment was announced. The writer, accepting the statements of his medical confrère with the utmost loyalty, felt that here at last was reasonable ground of hope for his patient. He took the medical paper to her house, and read to her the striking account of cure after cure effected by the new remedy. She instantly became filled with hope and eagerness, which was, indeed, the object of reading to her the remarkable stories of the cures which had been made. An experiment was begun with her under the most favourable possible circumstances. The curative drug was obtained, regardless of cost, from the purest sources; devoted nurses attended to the patient night and day; and the doctor could not have been more watchful and anxious had his own life been at stake instead of the patient's. For one week, for two, for even three or four, the general health markedly improved, the sufferings of the patient as decidedly diminished, and hope rose high in all hearts of a triumphant cure. But it was not so to be. The drug was an effective tonic, but had no other action. The hope that had animated the patient and her family had roused her to endure pains, to take food, and to make every effort to secure regular sleep. And so there had followed an all too brief modification of the worst symptoms—nothing more. In the course of a little more than a year the patient died, in spite of the best efforts that could be made on her behalf. Not only was the particular drug tried, of which we have spoken, but all kinds of orthodox and heterodox books were ransacked, and every method of treatment experimented with which offered the least show of possibility of being of service. The result was what has been stated.

There is no reason to doubt that hundreds of medical men who had cancer cases on hand at the time did exactly the same as the writer in trying the new drug. In fact, the whole medical profession, both at hospitals and in private practice, joined in an immediate series of experiments. What was the final result to doctors and to patients of all those testings and provings? It was this: In the course of three years the much-lauded drug was proved to be an entire delusion as a cancer curer; and it went as completely out of use as if it had never been brought into notice. Now what readers ought in all fairness to see is, that modern medical men at any rate are not averse to new methods, but that, on the contrary, they are always anxiously on the look out in every department of science and common experience for new remedies and new cures.

But it may be said that the introducer of the drug we have spoken of was a regular medical man, and that therefore his claims received full and immediate attention. Count Mattei, on the other hand, is not a medical man, and for that reason he is given the cold shoulder by the profession. But Pasteur is not a medical man; and yet it is he of all men whom the doctors of this country most delight to honour at the present moment. The plain truth about the matter is simply this, that we want cures, not mere reports of cures. If Count Mattei has remedies which will really cure, what is to prevent him gathering together a hundred cases of cancer, duly attested as such by medical men, into a hospital, and there treating them? At the end of three, six, or twelve months let him show them cured and well like other people. Or, since Count Mattei is represented in this country by loyal followers, what is to prevent any one of them—say Dr. Kennedy or Lady Paget—from gathering together a hundred *bona fide* and duly attested cases into a house or hospital, and showing them perfectly cured at the end of three, six, or twelve months? There will be no difficulty in securing a medical commission to watch the cases. If relays of doctors will spend days and nights in watching fasting men at the Aquarium, how much more willingly would they devote themselves to the demonstration and establishment of a method of cure which would save tens of thousands of lives in a single year? We ourselves would gladly undertake the formation of such a commission, and pay all the necessary expenses connected therewith, if there were the least possibility of its being demanded. As showing how ready doctors are to investigate the asserted cures by outsiders, as well as by regular practitioners, it is only necessary to recur again to M. Pasteur. In his case a commission was appointed, consisting of some of the most eminent scientists of this country. They went to Paris; they saw Pasteur and his work: and they were satisfied.

There is no more reason in the nature of things why a layman should not cure disease if he can than there is why General Booth's captains should not preach, or why an untrained draper's assistant should not hit the biggest cricketing score of the season if he is able. The whole world is open to all men to do what they can in it. To do; but not to merely pretend to do. He who can do can show what he has done. The man who requires to use laboured arguments in order to convince people of the actuality of his work invites suspicion. If Dr. Kennedy, following Count Mattei, can really cure cancer, he will be a wise man if he does not write another word to any public print on the subject. Instead of writing he will get together a number of undoubted cancer cases; he will show them to doctors who are above suspicion; he will cure them under the eyes of those doctors; and the doctors will become his willing trumpeters. If he does not do this we shall believe that it is because he cannot. If instead of doing it he continues to argue about it in the public press, we shall suspect him of sinister motives. Red tape and irrational conventionalism are stupid and ridiculous; but a want of candour and an unwillingness to submit to simple experimental tests smack strongly of knavery.

